# Appearance-Based Sequential Robot Localization Using a Patchwise Approximation of a Descriptor Manifold

**DOI:** 10.3390/s21072483

**Published:** 2021-04-02

**Authors:** Alberto Jaenal, Francisco-Angel Moreno, Javier Gonzalez-Jimenez

**Affiliations:** Machine Perception and Intelligent Robotics Group (MAPIR), Department of System Engineering and Automation Biomedical Research Institute of Malaga (IBIMA), University of Malaga, 29071 Málaga, Spain; ajaenal@uma.es (A.J.); javiergonzalez@uma.es (J.G.-J.)

**Keywords:** appearance-based localization, computer vision, Gaussian processes, manifold learning, robot vision systems, indoor positioning, image manifold, descriptor manifold

## Abstract

This paper addresses appearance-based robot localization in 2D with a sparse, lightweight map of the environment composed of descriptor–pose image pairs. Based on previous research in the field, we assume that image descriptors are samples of a low-dimensional Descriptor Manifold that is locally articulated by the camera pose. We propose a piecewise approximation of the geometry of such Descriptor Manifold through a tessellation of so-called *Patches of Smooth Appearance Change* (PSACs), which defines our *appearance map*. Upon this map, the presented robot localization method applies both a Gaussian Process Particle Filter (GPPF) to perform camera tracking and a Place Recognition (PR) technique for relocalization within the most likely PSACs according to the observed descriptor. A specific Gaussian Process (GP) is trained for each PSAC to regress a Gaussian distribution over the descriptor for any particle pose lying within that PSAC. The evaluation of the observed descriptor in this distribution gives us a likelihood, which is used as the weight for the particle. Besides, we model the impact of appearance variations on image descriptors as a white noise distribution within the GP formulation, ensuring adequate operation under lighting and scene appearance changes with respect to the conditions in which the map was constructed. A series of experiments with both real and synthetic images show that our method outperforms state-of-the-art appearance-based localization methods in terms of robustness and accuracy, with median errors below 0.3 m and 6°.

## 1. Introduction

Visual-based localization involves estimating the pose of a robot from a query image, taken with an on-board camera, within a previously mapped environment. The widely adopted approach relies on detecting local image features (e.g., points, segments) [1,2] that are projections of 3D physical landmarks. Though this feature-based localization has achieved great accuracy in the last years [3,4,5], it presents two major drawbacks that hinder long-term localization and mapping: (i) lack of robustness against image radiometric alterations; (ii) inefficiency of 2D-to-3D matching against large-scale 3D models [6]. A much less explored alternative to feature-based localization consists in localizing the robot through the scene appearance, represented by a descriptor of the whole image. According to this framework, localization is accomplished by comparing the appearance descriptor against a map composed of descriptor–pose pairs, without any explicit model of the scene’s geometric entities [7,8]. This approach turns out to be particularly robust against perceptual changes and also appropriate for large-scale localization, as demonstrated by the fact that it is included in the front-end of state-of-the-art Simultaneous Localization and Mapping (SLAM) pipelines to perform relocalization and loop closure, typically in the form of Place Recognition (PR) [3,5].

The accuracy of appearance-based localization is, however, quite limited. Good results are reported only when the camera follows a previously mapped trajectory (i.e., in one dimension) [9,10,11] or when it is very close to any of the poses of the map [8,12]. In this work, we investigate whether localization based only on appearance can deliver *continuous* solutions that are accurate and robust enough to become a practical alternative to methods based on 3D geometric features. We restrict this work to planar motion, that is, we aim to estimate the camera pose given by its 2D position and orientation (3 d.o.f.).

To this end, we first assume that images acquired in a certain environment are samples of a low-dimensional Image Manifold (IM) that can be locally parameterized (or articulated) by the camera pose. This assumption has been justified by previous works [13,14], but only exploited under unrealistic conditions, where the IM was sampled from a fine grid of poses in the environment under fixed lighting conditions. This IM is embedded in an extremely high-dimensional space: $R^{H\times W\times 3}$, where *W* and *H* stand for the width and height of the images, respectively. Patently, working in such extremely high-dimensional space is not only unfeasible, but also impractical since it lacks radiometric invariance. That is, the IM of a given environment might change drastically with the scene illumination and the automatic camera accommodation to light (e.g., gain and exposure time). Thus, it is primarily to project the images (i.e., samples of the IM) to a lower-dimensional space with a transformation that also provides such radiometric invariances [15]. This projection can be carried out by encoding the image into a descriptor vector, hence obtaining a new appearance space, the Descriptor Manifold (DM), which is still articulated by the camera pose. In this paper, we leverage Deep Learning (DL)-based holistic descriptors [8,16] to project the IM into a locally smooth DM. We are aware that such smoothness is not guaranteed for the descriptors employed here, since this feature has not been explicitly taken into account in their design. This issue will be addressed in future work, but in the context of our proposal, the selected DL-based descriptors perform reasonably well under this assumption.

**Figure 1:**
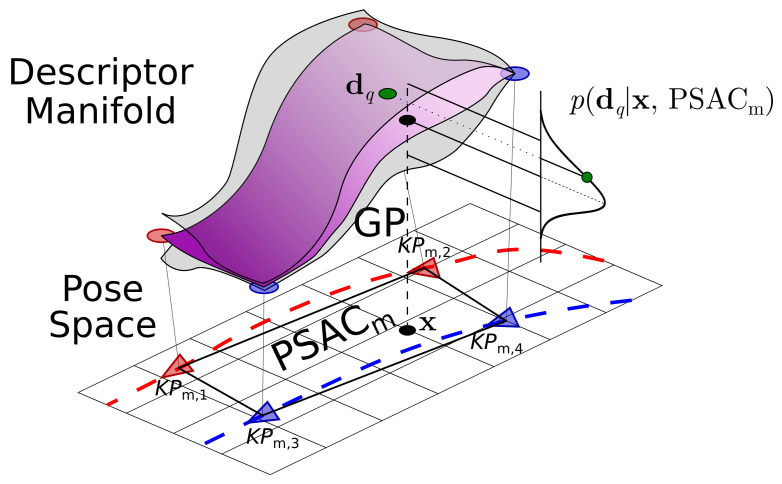
The Gaussian Process (GP) associated to a *Patch of Smooth Appearance Change* (PSACs) approximates the geometry of a neighborhood of the Descriptor Manifold (assumed to be locally smooth) with respect to the pose space, predicting the local likelihood $p(d_{q}|x,{\mathrm{PSAC}}_{m})$ of the observation $d_{q}$ in a given pose x. In this example, the descriptor–pose pairs are extracted from two previous trajectories of the robot (in red and blue).

Another capital aspect in appearance-based localization is that it requires an appropriate map, which, in our case, is built from samples of the DM that are annotated with their poses. In this paper, we assume that such samples, in the form of descriptor–pose image pairs, are given in advance and are representative of the visual appearance of the environment. Upon this set of pairs, we propose creating *Patches of Smooth Appearance Change* (PSACs), that is, regions that locally approximate the geometry of the DM using neighbor samples (see Figure 1). A tessellation of such PSACs results in a piecewise approximation of the DM that constitutes our *appearance map*, where pose data is only available at the vertices of the PSACs. The appearance smoothness within each PSAC allows us to accurately regress a descriptor for any pose within the pose space covered by the PSAC. This is accomplished through a Gaussian Process (GP), which delivers the Gaussian distribution of the regressed descriptor (refer to Figure 1).

Our proposal solves continuous sequential localization indoors by tracking the robot pose using a Gaussian Process Particle Filter (GPPF) [17,18] within the described *appearance map*. The particles are propagated with the robot odometry and weighted through the abovementioned Gaussian Process, which is implemented as the GPPF observation model for the image descriptor.

Pursuing to improve the robustness of our method against appearance changes, we model the descriptor variations in such situations as a white noise distribution that is introduced into the estimation of the observation likelihood. Finally, it is worth mentioning that our proposal can easily recover from the habitual PF particle degeneracy problem by launching a fast and multihypothesis camera relocalization procedure through global Place Recognition.

Our localization system has been validated with different indoor datasets affected by significant appearance changes, yielding notable results that outperform current state-of-the-art techniques, hence demonstrating its capability to reduce the gap between feature-based and appearance-based localization in terms of accuracy, while still leveraging the invariant nature of holistic descriptors.

## 2. Related Work

This section reviews two concepts that are essential for the scope of this work: Global image descriptors and Appearance-based localization.

### 2.1. Global Image Descriptors

A well-founded way of getting a consistent dimensionality reduction from the Image Manifold to the Descriptor Manifold is through *Manifold Learning* tools, like LLE [19] or Isomap [20]. Their performance, however, is limited to relatively simple IMs that result from sequences of quasi-planar motions or deformations, like face poses, person gait, or hand-written characters [21]. Unfortunately, images taken in a real 3D scene give rise to complex, highly twisted IMs, which also present discontinuities due to occlusion borders [22]. Moreover, typical *Manifold Learning* tools are hardly able to generalize their learned representations to images captured under different appearance settings [15]. This prevents their application to generating low-dimensional embeddings adequate for camera localization.

Nevertheless, Deep Learning (DL)-based holistic descriptors have recently proven their suitability to enclose information from complete images, effectively reducing their dimensionality, while adding invariance to extreme radiometric changes [8,12,23,24]. This feature has made DL-based descriptors highly suitable for diverse long-term robot applications [9,25], e.g., Place Recognition (PR), where the goal is to determine if a certain place has been already seen by comparing a query image against a lightweight set of images [26]. In addition, these descriptors have proven to more sensitively reflect pose fluctuations than local features [26], which is described, for example, by the *equivariance* property [27,28]. Since we are targeting robust robot operation under different appearance conditions, global descriptors arise as the natural choice to address appearance-based localization.

### 2.2. Appearance-Based Localization

Appearance-based localization is typically formulated as a two-step estimation problem: first, PR is performed to find the most similar images within the map and, subsequently, the pose of the query image is approximated from the location of the retrieved ones [29,30]. In this scenario, DL-based works have proposed to improve the second stage through Convolutional Neural Network architectures that estimate relative pose transformations between covisible images [31,32,33].

The addition of temporal and spatial sequential information to appearance-based localization methods based on single instances provides more consistency to the estimation of the pose, as it reduces the possibility of losing camera tracking due to, for instance, perceptual aliasing [26]. Following this idea, SeqSLAM [10,34] proposes a sequence-to-sequence matching framework that reformulates PR with the aim to incorporate sequentiality, leading to substantial improvements under extreme appearance changes. Building upon SeqSLAM, SMART [11] integrates odometry readings to provide more consistent results. More recently, *Network-Flow*-based formulations have also been proposed to solve appearance-based sequence matching under challenging conditions, addressing camera localization [35,36] or position-based navigation and mapping [37]. Despite their relevant results, the nature of all these works is discrete, unlike our proposal, restricting all possible estimation to the locations present on the map.

Conversely, CAT-SLAM [38] employs image sequentiality as a source of topometric information to improve the discrete maps used by FAB-MAP [9], allowing interpolation within the sequence map through the association of continuous increments on appearance and pose. Although the estimates produced by this approach are continuous, they are restricted to the mapped trajectory. Our work overcomes this constraint by requiring multiple sequences or pose grids as a source for constructing the map PSACs. This way, we can perform localization even at unvisited map locations near the PSACs, achieving, consequently, more accuracy and reliability.

An interesting alternative to pose interpolation is the use of Gaussian Processes (GPs) regression [39], nonparametric, general-purpose tools that allow generalizing discrete representations to a continuous model, and, hence, can be adapted to perform continuous localization within discrete maps. For instance, [40,41] employed GPs to generate position estimates for omnidirectional images in indoor maps, achieving good performance although lacking applicability to robot rotations. Our approach is instead designed to work with both 2D positions and rotations for conventional cameras.

In turn, the authors of [18] proposed Gaussian Process Particle Filters (GPPFs) to solve appearance-based localization in maps of descriptor–pose pairs. The GP works as the observation model of the PF, estimating the likelihood of the observed holistic descriptor at each of the particle positions. This localization pipeline was later improved in [42] by using only the nearest map neighbors in the GP regression, allowing efficient localization within large environments. Despite being promising, both works have three major drawbacks: (i) they define a unique Gaussian Process between poses and descriptors for the whole environment, assuming that the manifold geometry has a similar shape for the entire environment, thus leading to inaccurate estimations, (ii) they do not propose a relocalization process in case of losing tracking, and (iii) they only consider localization under the same appearance than the map, lacking robustness to radiometric alterations.

Inspired by these works, we employ a GPPF to solve appearance-based localization in a continuous and sequential fashion within challenging indoor environments. We solve their first problem by locally modeling the mapping between poses and descriptors via specific GPs for each PSAC, providing refined estimates for each neighborhood. Our proposal solves the second issue through a fast and multihypothesis relocalization process based on global PR within the map. Finally, the last issue is addressed by incorporating a model of the appearance variation between the mapped and query images to the map.

## 3. System Description

This section describes our proposal for the process of appearance-based camera localization. First, we define the elements that form the *appearance map*, which are key contributions in this work, and then we address PR-based localization and camera tracking using a probabilistic formulation based on a GPPF.

### 3.1. Patches of Smooth Appearance Change

The *Patches of Smooth Appearance Change* (PSACs) are regions that locally model the interrelation between camera poses and image descriptors, and represent areas where the change in appearance is small.

#### 3.1.1. Definition

The basic building unit of a PSAC is the pair $p_{i}=\left(d_{i},\;x_{i}\right)$, composed by the global descriptor $d_{i}\in R^{d}$ of an image and the pose $x_{i}\in SE\left(2\right)$ where it was captured.

**Figure 2:**
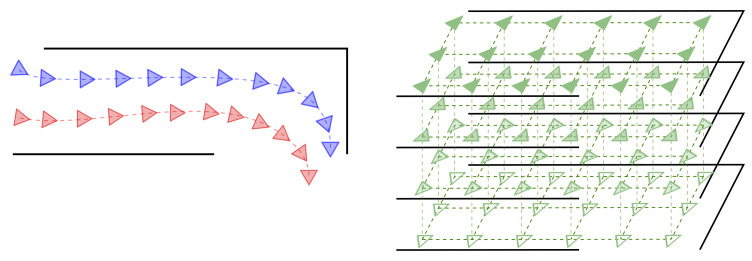
Each PSAC is constructed from descriptor–pose pairs that can be obtained from two different robot trajectories (left, blue and red) or a grid of poses (right, green). This is better seen in color.

We assume that these pairs are extracted from any of these two environmental representations (Figure 2): either from several **robot navigation sequences** (at least two) or from **pose grids** where the cameras have densely sampled the environment given fixed position and rotation increments (i.e., a regular grid). Optimally, a subset of these pairs should be selected so that they constitute the smallest number of samples from which the Descriptor Manifold (DM) can be approximated with sufficiently good accuracy. These *key* samples can be viewed as the equivalent to the *key-frames* in traditional, feature-based visual localization, and hence, we denote them *key-pairs* (*KPs*). Since determining such an optimal subset is a challenging issue itself, out of the scope of this work, the *KPs* are constantly sampled from the total collection of pairs.

Each PSAC is built from a group of adjacent *KPs* and approximates the DM in the region that they delimit. As explained later, the robot localization takes place within these PSACs by defining a suitable observation model for the GPPF (refer to Figure 1). Formally, let the *m*-th PSAC be

(1) $${\mathrm{PSAC}}_{m}=\left(\left\{KP_{m,i}|\;i=1,\ldots ,Q\right\},GP_{m}\right),$$

where Q≥3 is the number of *KPs* forming the PSAC. In turn, $GP_{m}$ is a Gaussian Process specifically optimized for that particular PSAC that delivers a Gaussian distribution over the image descriptor for any pose nearby the PSAC (further explained in Section 3.1.2).

Thus, in order to determine the closeness between a query pair $p_{q}=\left(d_{q},\;x_{q}\right)$ and a particular PSAC, we define two distance metrics as follows:

- The *appearance distance* $D_{m,q}^{a}$ from the query descriptor $d_{q}$ to the *m*-th PSAC is defined as the average of the descriptor distances to each of its constituent *key-pairs*:
  (2) $$D_{m,q}^{a}=D^{a}\left({\mathrm{PSAC}}_{m},d_{q}\right)=\frac{1}{Q}\sum_{i}^{Q}\left|\right|d_{q}-d_{m,i}{\left|\right|}_{2}.$$
- Similarly, but in the pose space, we define the *translational distance* $D_{m,q}^{t}$ from $x_{q}$ to the *m*-th PSAC as
  (3) $$D_{m,q}^{t}=D^{t}\left({\mathrm{PSAC}}_{m},x_{q}\right)=\frac{1}{Q}\sum_{i}^{Q}\left|\right|t_{q}-t_{m,i}{\left|\right|}_{2},$$
  with $t_{q}$ being the translational component of the pose $x_{q}=\left(t_{q},\theta_{q}\right)$.

Finally, the set of all PSACs covering the environment that has been sampled forms the **appearance map** M:

(4) $$M=\{{\mathrm{PSAC}}_{m}|\;m=1,\ldots ,M\},$$

with *M* being the number of PSACs. This way, we achieve a much more accurate approximation of the relation between the pose space and the DM within each PSAC, ultimately modeling in M, patchwise, the complete shape of the DM.

#### 3.1.2. GP Regression

GPs are powerful regression tools [39] that have previously demonstrated their validity as observation models in Particle Filters [17,18,42].

In this work, we learn a specific GP for each PSAC from its vertices *KPs* and also using all the nearest pairs, in terms of translational distance. Then, for a certain query pose $x_{q}$, the $GP_{m}$ delivers an isotropic Gaussian distribution $N(\mu_{m,q},\sigma_{m,q}^{2}I_{d})$, where $\mu_{m,q}\in R^{d}$ and ${\sigma^{2}}_{m,q}\in R$ stand for its mean and uncertainty, respectively. This distribution is finally employed to estimate the likelihood $p(d_{q}|x_{q},{\mathrm{PSAC}}_{m})$ of an observed image descriptor $d_{q}$, given the query pose $x_{q}$ within the ${\mathrm{PSAC}}_{m}$.

For this, the GP regression employs a *kernel* *k*, which measures the similarity between two input 2-D poses $(x_{i}$, $x_{j})$, with this structure:

(5) $$k\left(x_{i},x_{j}\right)=k_{RBF}\left(t_{i},t_{j}\right)\;\cdot \;k_{RBF}\left(\theta_{i},\theta_{j}\right)+k_{W}\left(x_{i},x_{j}\right).$$

**Figure 3:**
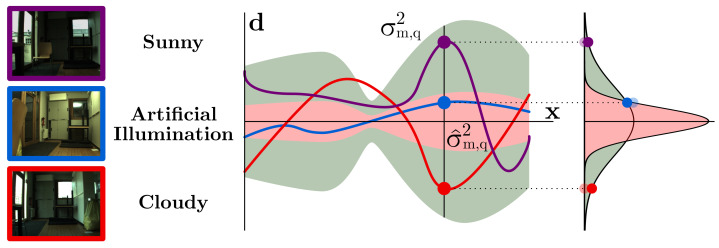
These three images have been captured at the same pose but with different appearances. Ideally, their descriptors (blue, red, and purple dots) should be identical but, in practice, certain inaccuracies appear. As the GP learns the descriptor distribution uniquely from the appearance of the map, this variation is not considered, leading to an underestimated GP uncertainty (red area, ${\hat{\sigma }}_{m,q}^{2}$). The inclusion of white noise expands such uncertainty (green area, $\sigma_{m,q}^{2}$), solving this issue.

This *kernel k* first multiplies two Radial Basis Function (RBF) *kernels* $k_{RBF}\left(a_{i},a_{j}\right)=\beta_{a}\;\exp(-\alpha_{a}\left|\right|a_{i}-a_{j}{\left|\right|}_{2}^{2})$ ($\alpha_{a}$ and $\beta_{a}$ are optimizable parameters) for the separated translational and rotational components of the evaluated poses x=(t,θ). Then, a White Noise *kernel* $k_{W}\left(a_{i},a_{j}\right)=\sigma_{W}^{2}\delta \left(a_{i}-a_{j}\right)$ is added, which models the variation suffered by the image descriptors taken at the same pose but under different appearances (refer to Figure 3). This is justified because, although global PR descriptors have demonstrated outstanding results in terms of invariance, such invariance is not ideal and small differences might appear. Thereby, since the construction of the map M is typically carried out considering just one particular appearance, and we aim for the robot localization to be operational under diverse radiometric settings, we propose the inclusion of a white noise distribution accounting for this circumstance in the regression. We model such descriptor variation with the variance $\sigma_{W}^{2}$, computed as the average discrepancy between the descriptor variances of pose adjacent pairs $p_{i}$, under the same $\sigma_{i,\mathrm{same}}^{2}$ and different $\sigma_{i,\mathrm{diff}}^{2}$ illumination settings:

(6) $$\sigma_{W}^{2}=\frac{1}{N}\sum_{i}^{N}\left(\sigma_{i,\mathrm{diff}}^{2}-\sigma_{i,\mathrm{same}}^{2}\right).$$

### 3.2. Robot Localization

Once we have defined all the elements involved in the representation of the environment, we address here the process of localization within the appearance map M. We aim to estimate the robot pose through appearance-based continuous tracking using a Gaussian Process Particle Filter (GPPF), namely, a PF that employs the GPs described above as observation models. Being well-known robot localization tools, we do not provide a deep description of Particle Filters here but instead refer to the reader to the seminar work [43] for further information.

At time-step *t*, each of the *P* particles in the filter represent a robot pose estimation $x_{p}^{\left(t\right)}$ with an associated weight $w_{p}^{\left(t\right)}$, proportional to its likelihood. Besides, each particle is assigned to a certain region ${\mathrm{PSAC}}_{m,p}^{\left(t\right)}$, as explained next.

#### 3.2.1. System Initialization

When the PF starts, we perform global localization based on Place Recognition to select the most similar PSAC in M to the query descriptor according to its appearance:

(7) $${\mathrm{PSAC}}_{\hat{m}}=\min_{{\mathrm{PSAC}}_{m}\in M}D_{m,q}^{a}.$$

To account for multihypothesis initialization, we also consider as candidates those PSACs whose *appearance distance* is under a certain threshold proportional to $D_{\hat{m},q}^{a}$. Subsequently, the particles are uniformly assigned and distributed among all candidate PSACs, setting their initial weights to $w_{p}^{\left(t_{0}\right)}=\frac{1}{P}$.

Note that, if the robot tracking is lost during navigation, this procedure is launched again to reinitialize the system and perform relocalization.

#### 3.2.2. Robot Tracking

Once each particle is assigned to a candidate PSAC, the robot pose estimation is carried out following the traditional *propagation-weighting* sequence:

**Propagation**. First, the particles are propagated according to the robot odometry:

(8) $$x_{p}^{\left(t\right)}=x_{p}^{\left(t-1\right)}⊕\upsilon^{\left(t\right)},$$

with $\upsilon^{\left(t\right)}∼N\left({\bar{\upsilon }}^{\left(t\right)},\Sigma_{\upsilon }\right)$ representing noisy odometry readings, and ⊕ being the pose composition operator in SE(2) [44].

**Weighting**. After the propagation, the *translational distance* between each particle’s pose and all the PSACs is computed, so that the particle is assigned to the nearest PSAC (${\mathrm{PSAC}}_{m,p}^{\left(t\right)}$). Then, we use the GP regressed in the PSAC to locally evaluate the likelihood of the observed descriptor $d_{q}$ at the particle pose $x_{p}^{\left(t\right)}$ as follows:

(9) $$w_{p}^{\left(t\right)}=p\left(d_{q}|x_{p}^{\left(t\right)},{\mathrm{PSAC}}_{m,p}^{\left(t\right)}\right)∼\exp\left(-\frac{d}{2}\ln\left({\sigma^{2}}_{m,p}^{\left(t\right)}\right)-\frac{\left|\right|d_{q}-\mu_{m,p}^{\left(t\right)}{\left|\right|}_{2}^{2}}{2({\sigma^{2}}_{m,p}^{\left(t\right)})}\right),$$

with *d* being the dimension of the descriptor.

Finally, apart from *propagation* and *weighting*, two more operations can be occasionally applied to the particles.

**Resampling:** In order to prevent particle degeneracy, the GPPF resamples when the number of effective particles is too low, promoting particles with higher weights.

**Reinitializing:** During normal operation, the GPPF may lose the tracking of the camera, mainly due to extremely challenging conditions in the images (e.g., very strong change appearances, presence of several dynamic objects). We identify this situation by inspecting the *translational distance* between each particle and the *centroid* of its assigned PSAC, defined as the average pose of all the key-pairs forming the PSAC. If all particles are at least twice farther from the centroid of their assigned PSAC than its constituent *key-pairs*, the tracking is considered lost. Consequently, the PF relocalizes by following the aforementioned initialization procedure.

## 4. Experimental Results

In this section, we present three experiments to evaluate the performance of our appearance-based localization system.

First, we carry out a verification of the regression outcome in Section 4.1, with the aim to experimentally validate the hypothesis of a smooth Descriptor Manifold within the regions covered by each PSAC. In Section 4.2 and Section 4.3, we test our proposal with four different state-of-the-art global descriptors in two different datasets, with a combination of setups for the map sampling. This has provided us with an insight about the error incurred by our proposal and has allowed us to determine the best configuration for localization. The second experiment, in Section 4.4, compares the resulting setup with three appearance-based localization alternatives in terms of accuracy and robustness, revealing that our system equals or improves their performance in every scenario.

It is important to highlight that this evaluation does not include comparisons with feature-based localization methods, since they are not compatible with appearance changes in the images used for both mapping and localization, which is one of the main benefits of our proposal.

**Figure 4:**
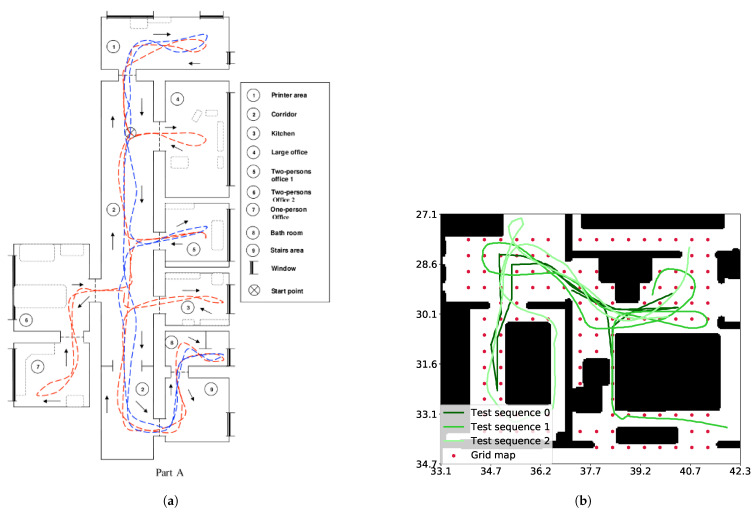
Environments and sequences employed for evaluation. (**a**) Map of the COLD-Freiburg Part A environment. Samples of the standard and extended routes are depicted in blue and red, respectively (image from [45]). (**b**) Map of the house rendered by the SUNCG environment (the house employed was *034e4c22e506f89d668771831080b291*). The dense grid poses are shown in red and the test sequences in different shades of green. Black regions depict objects, where the robot is not able to navigate.

We employed two different indoor datasets for the experiments:

- The **COLD-Freiburg database** [45], which includes real images from an office gathered with a mobile robot under different appearance conditions. We have used only a representative subset of the sequences in *part A* of the dataset (Figure 4a).
- The synthetic **SUNCG Dataset** [46] rendered through the virtual **HOUSE3D** environment [47] allows us to test the localization on a grid map (see Figure 4b).

In turn, regarding the global image representation, we have tested the following state-of-the-art appearance descriptors:

- **NetVLAD**: VGG16 [48] based on off-the-shelf, 4096-sized NetVLAD features with Principal Component Analysis (PCA) whitening [8].
- **ResNet-101 GeM**: ResNet-101-based [49] fine-tuned generalized-mean features with learned whitening [12].
- **1M COLD Quadruplet** and **1M RobotCar Volume**: end-to-end learned condition invariant features with VGG16 NetVLAD [24] with quadruplet and volume loss functions in two different datasets.

Although none of these descriptors has been specifically designed to fulfill suitable properties for our pose regression approach, they have achieved promising results in terms of localization accuracy, as shown next. We used the GPy tool [50] to implement the proposed Gaussian Processes and empirically determined $\sigma_{W}^{2}$ from Equation (6), for each descriptor, by randomly sampling N= 2000 adjacent pairs with diverse illumination settings from the COLD-Freiburg database.

In this evaluation, we employ as metrics the median errors in translation and rotation (to inspect our method’s accuracy), as well as the percentage of correctly localized frames (which illustrates the tracking and relocalization capabilities of our method). It is worth mentioning that traditional trajectory-based evaluation metrics as Absolute Trajectory Error (ATE) or Relative Pose Errors (RPE) are not applicable to this approach since our proposal, and appearance-based localization methods in general, yields global pose estimations that are not guaranteed to belong to a trajectory, due to possible tracking losses and relocalization situations.

### 4.1. Corridor: Sanity Check

The main assumption of our proposal is the hypothesis of a locally smooth Descriptor Manifold with respect to the pose, on which PSACs are based. Since this assumption is not justified by previous work, we have conducted a basic test to evaluate the regression outcome of the PSACs in a simple scenario.

The proposed experiment studies the evolution of the image descriptor along a simple, linear trajectory, by comparing the observed descriptor and the mean of the descriptor distribution resulting from the regression within the PSAC. In this manner, the behavior of the descriptor can be examined along the corridor axis in order to prove its continuity and the validity of the PSAC approximation. For this, we have selected a portion of an artificially illuminated (*night*) sequence where the robot traveled along a ∼8 m-long corridor, as well as the NetVLAD image descriptor. For the PSACs, we used a map constructed with images with the same appearance selected every 20 frames.

**Figure 5:**
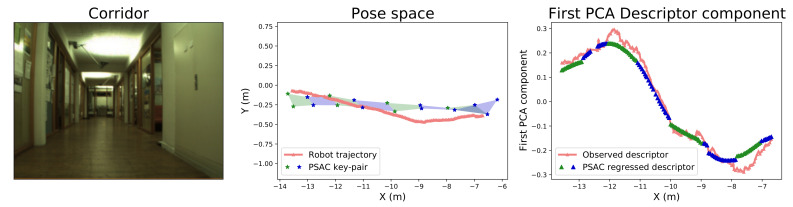
Experimental study of the global image descriptor behavior along a corridor of the COLD-Freiburg database (left image). The central figure depicts the robot pose trajectory and the PSACs (shadowed areas) through which it navigates. The rightmost figure compares, along the corridor, the behavior of the first Principle Component Analysis (PCA) component of the observed descriptor (coral) and the PSAC regression output (blue and green). This is better seen in color.

In order to represent the evolution of the descriptors, we have applied Principal Component Analysis (PCA) to them and represented the first PCA element (that with larger variation). Thus, Figure 5 depicts the trajectory of the robot through the corridor along with the value of said first PCA element for both the observed descriptor and the mean of the descriptor distribution regressed by the GPs at each PSAC. The displayed results demonstrate that the descriptor has a continuous evolution along the corridor, almost lineal in the central part. Besides, the PSACs are proved to also have a continuous outcome and to approximate very accurately the values of the observed descriptor along the sampled trajectory.

### 4.2. COLD: Sequential Map Testing

The COLD-Freiburg database (*part A*) provides odometry readings and real images for two different itineraries, namely, (i) **extended** (∼100 m-long), which covers the whole environment; (ii) **standard** (∼70 m), covering a subset of the environment, both depicted in Figure 4a. The dataset also provides images gathered under three different lighting conditions: at *night* (with artificial illumination), and on *cloudy* and *sunny* days.

In order to create the *appearance maps* for the experiments, we employed the first and second *night* sequences of the extended itinerary, since images captured under artificial illumination do not suffer from severe exposure changes or saturation like under the remaining conditions. From here on, we will refer to these as *map sequences*. Specifically, *key-pairs* from both *map sequences* have been obtained through Constant Sampling (CS) every 10, 20, and 30 pairs, resulting in three different maps with diverse density (described in more detail in Table 1), using Q= 4 *KPs* to construct every PSAC.

Finally, we have setup an extensive evaluation with six other sequences including different routes and illumination conditions: the first *night*; *cloudy* and *sunny* sequences of the **standard** part; the first *cloudy* and *sunny* sequences; and the third *night* sequence of the **extended** part.

**Figure 6:**
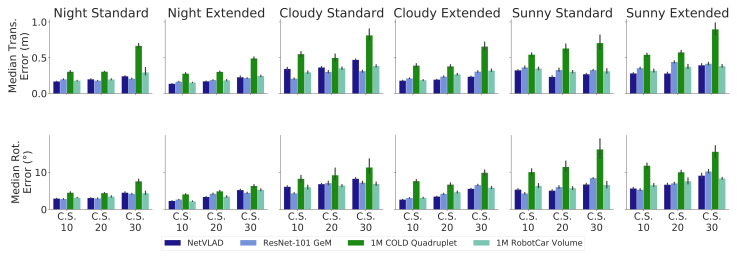
Comparison of the median translational and rotational errors of our proposal in the COLD-Freiburg dataset, tested at every sequence and with different Constant Sampling (CS) rates, using each holistic descriptors with 10^3^ particles. Note that the maps were constructed employing sequences under similar conditions to the *night* sequences. This is better seen in color.

Figure 6 shows a comprehensive test study depicting the localization performance of our proposal after twenty runs for all test sequences at each map, using the median translational (top) and rotational (down) errors as metrics. Note that the number of particles for the PF has been set to *P* = 10^3^, as we have empirically found that increasing that number does not improve the accuracy results. The overall performance shows a median error predominantly below 0.3 m and 6°, which denotes promising results given the pure appearance nature of our approach, i.e., with no geometrical feature employed for localization.

The results in Figure 6 show that scene appearance seems to be a key issue regarding the system’s accuracy, as our proposal achieves better results in less-demanding lighting conditions like artificial illumination (night) or cloudy. Nevertheless, our system still demonstrates notable performance under challenging radiometric conditions, such as in sunny sequences (e.g., presence of lens flares and image saturation), hence proving its suitability for robust appearance-based localization.

On the other hand, the number of *KPs* that form the map is another factor influencing the performance, since the PSACs approximate the pose–descriptor interrelation the closer their *KPs* are. Although not particularly significant under advantageous conditions, this factor severely affects performance under challenging situations, as in *sunny* sequences, where localization is hindered as the sampling frequency decreases. Note that a more elaborate mapping technique than CS would improve these results, since an optimal selection of *KPs* would conform PSACs that achieve a more precise description of the DM geometry. Nevertheless, this will be explored in future work while, in this paper, we rely on CS to get still nonoptimal but notable results.

Finally, regarding the tested PR descriptors, the results show that in most cases, all perform similarly, with NetVLAD mostly achieving slightly better results. In turn, 1M COLD Quadruplet seems to struggle under complex illumination conditions, which might indicate that its empirically estimated white noise variance is unlikely to account adequately for these cases. The similar performance shown by all descriptors agrees with the fact that none of them has been specifically trained for appearance-based localization.

### 4.3. SUNCG: Grid Map Testing

The SUNCG Dataset provides a set of synthetic houses where a virtual camera can be placed at any pose. This feature allowed us to create a regular grid map in the space of planar poses with the camera and then to evaluate the impact of the map density in our proposal. Note that this dataset does not present appearance changes, and hence, the effect of such a characteristic cannot be evaluated in this experiment.

First, our *dense* grid map was created by selecting *KPs* with constant increments of 0.5 m in translation and 36° in rotation (refer to the red dots in Figure 4b). Then, we used subsampling to generate more grid maps for the evaluation, as described in Table 1, namely, the *sparse-position map* (subsampling half of the positions); the *sparse-rotation map* (subsampling half of the orientations); and the *sparse-position-rotation map* (subsampling both at the same time). In this case, we created PSACs with Q=8 *KPs* for all maps. Additionally, we have recorded three ∼30 m-long test sequences following the trajectories shown in shades of green in Figure 4b, generating a *synthetic odometry* corrupted by zero-mean Gaussian noise with $\sigma_{u}$ = (0.06 m, 1°).

**Figure 7:**
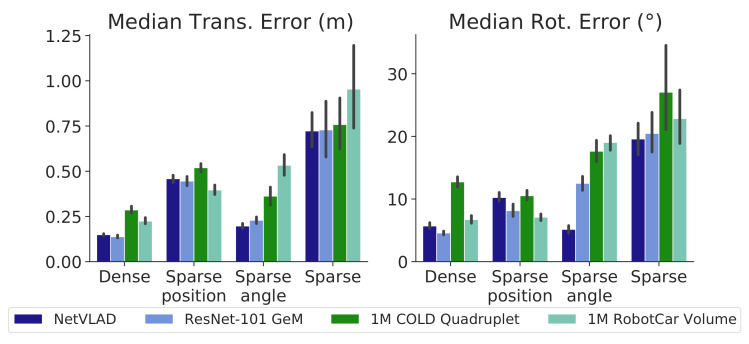
Median tracking errors of our proposal for the test sequences on the SUNCG generated maps, with 10^3^ particles.

The results of this experiment are shown in Figure 7, comparing the median errors in translation (left) and rotation (right) for all the descriptors employed in the previous experiment and for the described versions of the grid map. Again, we have set *P* = 10^3^ particles for the PF. As can be seen, our proposal yields median errors under 0.2 m and 6 in the *dense map*, while using subsampled maps hinders the process of localization. It can be noted that subsampling exclusively on rotations does not worsen the accuracy, while subsampling positions has a noticeable impact on the overall performance. Consequently, PSACs prove to handle information sparseness more efficiently in orientation than in position. Not surprisingly, subsampling in both position and orientation clearly achieves the worst localization performance due to the combined loss of information.

Regarding the holistic descriptors, all of them again demonstrate a similar behavior for each subsampling case, with 1M RobotCar Volume performing worst. ResNet-101 GeM and NetVLAD, in turn, achieve the best performance.

These results demonstrate that uniform grid sampling is a rough strategy for mapping environments, achieving results highly dependent on the sampling density. Besides, the construction of such maps with real robots becomes largely time-consuming, mostly being realizable when using virtual environments. Future work should investigate more elaborated strategies, designed to fulfill more adequate criteria concerning the map creation, ultimately pursuing an optimal approximation of the DM geometry.

### 4.4. Comparative Study

Finally, we compare the localization performance between our proposal and state-of-the-art appearance-based methods of diverse nature in both datasets. For the setup of our method, we selected the NetVLAD descriptor due to its performance against appearance changes, and added the *KPs* every 20 pairs for the COLD dataset, as it represents a fair trade-off between accuracy and the number of *KPs* employed.

These are the appearance-based localization methods involved in the comparison:

- **Gaussian Process Particle Filter (GPPF)** [42], configured with *P* = 10^3^ particles.
- The **Pairwise Relative Pose estimator (PRP)** presented in [31]: a CNN-based regressor that estimates the pose transform between the query and the 5 most similar map images obtained through PR.
- The **Network flow** solution proposed in [37]: a sequential sparse localization method that includes *uniform* and *flow-based* mapping, both considered in this study. In order to make the results comparable, we modified its outcome, which is sparse, to produce continuous estimations. For that, we used the following weighting after the bipartite matching:
  (10) $$x_{i}=\frac{\sum_{j}^{N_{k}}\frac{y_{i}}{y_{ij}}\;x_{i}}{\sum_{j}^{N_{k}}\frac{y_{i}}{y_{ij}}}\;$$
  where $x_{j}$ is the pose of the each of the $N_{k}=5$ most contributing *KPs*, $y_{ij}$ is the flow connecting the *i*-th query and the *j*-th *KP*, and $y_{i}=\sum_{j}^{N_{k}}y_{ij}$ represents the query flow from the nearer *KPs* (refer to [37] for further info).
- **Our approach**, configured with *P* = 10^3^ particles.

Table 2 shows the compared performance between all the described algorithms after twenty runs for each scenario. Note that, apart from the median errors, we included the percentage of correctly localized frames along the trajectory, showing the tracking robustness and relocalization potential. Concretely, a frame is considered to be correctly localized when the distance between the estimate and its true pose is below (0.5 m, 10°).

As can be seen, the challenging radiometric conditions in the COLD-Freiburg database caused the GPPF method to lose tracking, while the PRP estimator achieved low accuracy as a result of not exploiting the trajectory sequentiality, performing PR at every time-step instead. In turn, the solutions based on Network flow provide very accurate estimations in general, with the best results achieved by the uniformly sampled map under favorable conditions (i.e., *night* and *cloudy* sequences) and slightly worse in the case of severe appearance changes (i.e., *sunny* sequences). Our proposal, in contrast, demonstrates providing consistent results regardless of the appearance settings, achieving similar results to the Network flow solution in favorable conditions and outperforming all other methods under challenging radiometric settings.

In the case of the SUNCG dataset, the formulation proposed by the Network flow is incompatible with grid maps covering multiple rotations at the same location, as they are conceived to work only with positions. In turn, PRP and GPPF obtain low performance, even worsened in subsampled maps, while our proposal achieves the best results.

Despite its similarity with our approach, GPPF has shown to be unable to achieve robust localization due to the abovementioned issues: (i) deficiencies from considering a single pose–descriptor mapping for the whole environment, (ii) the absence of a relocalization process, and (iii) its limitation to environments without radiometric changes.

In conclusion, the presented comparison proves that these state-of-the-art localization methods based on appearance cannot provide both consistent and accurate localization estimations while operating within maps of diverse nature and captured under different appearance conditions. Our method, in turn, achieves higher performance in these conditions in terms of precision and robustness, showing notable results given its pure appearance nature.

## 5. Conclusions

We have presented a system for appearance-based robot localization that provides accurate, continuous pose estimations for camera navigation within a 2D environment under diverse radiometric conditions. Our proposal relies on the assumption that image global descriptors form a manifold articulated by the camera pose that adequately approximates the Image Manifold. This way, we gather pose–descriptor pairs from a lightweight map in order to create locally smooth regions called *Patches of Smooth Appearance Change* (PSACs) that shape, piecewise, the Descriptor Manifold geometry. Additionally, we robustly deal with appearance changes by modeling the descriptor variations under a white noise distribution.

We implemented a sequential camera tracking system built upon a Gaussian Process Particle Filter, which allows for multihypothesis pose estimation. Thus, our system optimizes a specific GP for each PSAC, subsequently being employed to define a local observation model of the descriptor for the Particle Filter. Furthermore, our method includes a relocalization process based on PR in case of tracking loss.

A first set of experiments has shown our proposal’s error baseline in different environments and for a selection of holistic descriptors, revealing the most suitable configuration for our system. Finally, we have presented a comprehensive evaluation of the localization performance, showing that our approach outperforms state-of-the-art appearance-based localization methods in both tracking accuracy and robustness, even using images with challenging illuminations, yielding median errors below 0.3 m and 6°. Consequently, we have proven that pure appearance-based systems can produce continuous estimations with promising results in terms of accuracy, while working with lightweight maps and achieving robustness under strong appearance changes.

Future work includes research about (i) building the appearance map in an optimal way and wisely selecting where to sample the Descriptor Manifold; (ii) the design of a novel holistic descriptor that is more adequate to perform pose regression while keeping high invariance to radiometric changes.

## Figures and Tables

**Table 1:** Compared statistics of the created appearance maps. The dimension is calculated for a 2048-sized global descriptor.

**Dataset**	**Area**	**Map**	***Key-Pairs***	**PSACs**	**Size**	**Construction**
			(***KPs***)		**(Mb)**	**Time (s)**
COLD Database	∼900 m${}^{2}$	Samp. 10	559	321	2.18	56.68
		Samp. 20	280	159	1.09	32.27
		Samp. 30	187	103	0.73	25.92
SUNCG	∼45 m${}^{2}$	Dense	1203	679	4.69	140.94
		Sparse pos	451	227	1.76	129.36
		Sparse rot	723	432	2.82	136.95
		Sparse pos-rot	271	149	1.05	107.93

**Table 2:** Comparative median position and rotation errors, and % of correctly localized frames (*m*, °, (%)) of different state-of-the-art appearance-based localization methods. A frame is correctly localized when the distance between the estimate and its true pose is below (0.5 m, 10°) (L: sequences where the tracking got lost. N/A: not applicable). PRP—Pairwise Relative Pose estimator; PR—Place R.

Dataset	Map	Sequence	GPPF [18,42] +	PRP CNN [31] +	Network Flow [37] +	Network Flow [37] +	Our Method
			Unif. Sampl	NetVLAD PR	Unif. Sampl.	Flow Sampl	C.S.
COLD Database	Samp. 20	Night std	L	1.17, 10.94 (8%)	**0.19**, 4.33 (66%)	0.26, 4.66 (60%)	0.2, **3.08** (**85**%)
		Cloudy std	L	1.93, 14.59 (4%)	0.31, **4.76** (**57**%)	0.36, 5.29 (46%)	**0.3**, 5.82 (56%)
		Sunny std	L	2.2, 14.75 (3%)	0.36, 6.91 (40%)	0.42, 7.7 (33%)	**0.23**, **5.08** (**66**%)
		Night ext	L	1.27, 11.07 (8%)	0.22, 3.88 (69%)	0.26, 4.36 (61%)	**0.17**, **3.38** (**82**%)
		Cloudy ext	L	1.46, 12.17 (6%)	0.22, 4.13 (60%)	0.31, 5.12 (52%)	**0.2**, **3.48** (**82**%)
		Sunny ext	L	2.11, 16.59 (2%)	0.3, 6.95 (50%)	0.35, 8.14 (39%)	**0.28**, **6.67** (**54**%)
SUNCG	Dense	Test sequence	1.07, 6.07 (17%)	0.75, 12.14 (13%)	N/A	N/A	**0.15**, **5.69** (**60**%)
	Sparse pos		1.21, 9.16 (7%)	1.14, 19.76 (2%)	N/A	N/A	**0.46**, **4.30** (**51**%)
	Sparse rot		1.24, 12.36 (2%)	0.89, 19.76 (6%)	N/A	N/A	**0.20**, **5.15** (**57**%)
	Sparse pos-rot		1.62, 20.16 (0%)	1.51, 24.51 (1%)	N/A	N/A	**0.72**, **18.58** (**19**%)

## Data Availability

Not applicable.

## Abbreviations

The following abbreviations are used in this manuscript:

PSAC	Patch of Smooth Appearance Change
PR	Place Recognition
GP	Gaussian Process
GPPF	Gaussian Process Particle Filter
PF	Particle Filter
SLAM	Simultaneous Localization and Mapping
IM	Image Manifold
DM	Descriptor Manifold
DL	Deep Learning
KP	Key-pair
CS	Constant Sampling
